# Response of garlic (*Allium sativum* L.) bolting and bulbing to temperature and photoperiod treatments

**DOI:** 10.1242/bio.016444

**Published:** 2016-03-30

**Authors:** Cuinan Wu, Mengyi Wang, Zhihui Cheng, Huanwen Meng

**Affiliations:** College of Horticulture, Northwest A&F University, Yangling, Shaanxi 712100, China

**Keywords:** Bolting, Bulbing, Methyl jasmonates, Photoperiod, Phytohormone, Temperature

## Abstract

This research was conducted to evaluate the effect of temperature and photoperiod treatments on the bolting and bulb formation of three local garlic cultivars (cvs) in two consecutive years. Naturally vernalized plants of cvs G107, G025 and G064 were transplanted into growth chambers and subjected to various combinations of temperature [T_15/10_, 15°C/10°C; T_20/15_, 20°C/15°C and T_25/18_, 25°C/18°C (day/night)] and photoperiod (L_8_, 8 h and L_14_,14 h) treatments. Plant growth, endogenous phytohormone and methyl jasmonate (MeJA) levels, along with the bolting and yield of garlic were evaluated. The experimental results from two consecutive years indicated that higher temperature (20°C or 25°C) and longer photoperiod (14 h) treatments significantly enhanced the garlic bolting, bulbing and cloving with a shorter growth period and a higher bulb weight. Moreover, the endogenous phytohormone and MeJA levels in the test plants were significantly increased by the higher temperature (25°C for the phytohormone level; 20°C for the MeJA level) and longer photoperiod [14 h, except for abscisic acid (ABA), which had the highest level at 8 h] conditions and were decreased by the lowest test temperature (15°C) and shorter photoperiod (8 h, except for ABA) conditions. This response coincided with that of the bulbing index, bolting rate, growth period and bulb weight. In addition, plants treated under the conditions of 20°C/15°C–14 h and 25°C/18°C–14 h produced the highest phytohormone levels (except for ABA) for cvs G025 and G064, respectively, and showed the best bolting and bulbing behavior. It is reasonable to assume that endogenous phytohormone (especially gibberellic acid) and MeJA levels are highly related to garlic bolting and bulbing, which might lead to the different responses of the three studied cultivars to the combination of temperature and photoperiod treatments. Furthermore, cvs G107 and G025 bolt well and have better bulb formation under 20°C–14 h conditions, while the conditions of 25°C–14 h are critical for the bolting and bulbing of cv. G064.

## Abstract (precis)

**Summary:** We show that a higher temperature and longer photoperiod are essential for garlic bolting and bulbing. Varied endogenous phytohormone and MeJA levels might lead to the different responses of the studied cultivars to treatments.

## INTRODUCTION

Garlic (*Allium sativum* L.) is an asexually propagated crop with high morphological diversity (Bradley et al., 1996) and is favored for its culinary and medicinal uses throughout the world. Vernalization fulfillment, followed by a higher temperature and long photoperiod is indispensable for garlic growth and development (Wu et al., 2015). However, the uncertainty of specific parameters, the interaction between temperature and photoperiod and the mechanism for bolting and bulbing have been key restrictions for the developmental regulation of garlic or the design of cultivation seasons and systems. A thorough understanding of the effects of environmental conditions (temperature and photoperiod) on garlic development and the changes of endogenous phytohormone and methyl jasmonate (MeJA) levels during this process should improve our knowledge of the bolting and bulbing processes and facilitate the production of a year-round supply of fresh scape and bulb.

Garlic genotypes are categorized as non-bolting, semi-bolting, and bolting (Takagi, 1990; Kamenetsky and Rabinowitch, 2001; Etoh and Simon, 2002; Kamenetsky et al., 2004a,b) and differ considerably in bolting ability, scape length and seed production (Mathew et al., 2010). In bolting accessions, specific combinations of temperature and photoperiod significantly influence the reproductive processes (Mathew et al., 2010). Long-photoperiod conditions trigger the initial elongation of flower stalks (Kamenetsky et al., 2004a; Mathew et al., 2010). Meanwhile, the bulbing and cloving of garlic are influenced by the length of the day and the temperature to which the dormant cloves or growing plants are exposed before bulbing begins (Bandara et al., 2000). In general, low initial temperatures followed by long days are essential for bolting and the formation of bulbs and cloves (Kolev, 1962). However, the competition for resources by the simultaneously developing bulb and inflorescence sinks determines the fate of stalk elongation and bulbing (Etoh and Simon, 2002; Le Nard and De Hertogh, 1993). In onion bulbs, a strong sink in early bulb development stages suppresses the growth and differentiation of the young inflorescence with consequent drying out of the flower stalk (scape) (Mathew et al., 2010). Hence, it was proposed that the influence of the temperature and photoperiod on scape and bulb development should be considered in the background of the simultaneous but competitive development of storage (bulb) and reproductive (scape) organs in garlic (Mathew et al., 2010).

To date, the effect of temperature and photoperiod on bulb formation has been studied for some *Alliums* cultivars. Brewster (1977) found that the bulb formation of onion and its subsequent growth were influenced by temperature and photoperiod and bulbing was promoted by long days and high temperatures. Moreover, in some cultivars, bulbing only occurred when dual thresholds of a minimum thermal time of 600 degree days and a photoperiod of 13.75 h were reached (Lancaster et al., 1996). However, to the best of our knowledge, the critical conditions for bolting and bulbing and the change in the signal substance during this process within *A. sativum* have received limited research attention (except for Kamenetsky et al., 2004b and Mathew et al., 2010). The investigation of the effects of temperature and photoperiod on garlic development will provide insight into the mechanism of garlic bolting and bulbing and will offer environmental tools (adjustable temperatures and photoperiods) for developmental regulation.

The regulation of bolting and bulbing is composed of complicated processes created by an intricate network of signaling pathways. Long photoperiods are known to improve the levels of endogenous gibberellins, with consequent flower bud differentiation (Mathew et al., 2010; King et al., 2006). Many studies have shown that gibberellic acid (GA) could partially or fully replace vernalization for some plants (Chakravarti, 1958; Guo et al., 2004; Nyarko et al., 2007). Aung et al. (1969) observed an increase in the endogenous GA levels of long day or biennial plants during the process of floral induction. However, GA is considered an inhibitor of bulb formation (Koda and Okazawa, 1983; Xu et al., 1998). Li et al. (2000) studied the effect of exogenous GA on garlic and found that exogenous GA inhibited the increase of the scape and bulb yield. It was likely that GA did not act directly on the inhibition of bulbing, instead enhancing the activity of a ‘bulbing inhibition substance’ (Langens-Gerrits et al., 2003; Le Guen-Le Saos et al., 2002; Wang and Guo, 2002). Abscisic acid (ABA) generally plays an important role in plant defense against biotic or abiotic stresses. It was assumed that ABA acts similarly to GA in the early stage of plant bolting (Song et al., 2007). Su et al. (2007) indicated that endogenous ABA levels of Welsh onion (*Allium fistulosum* L.) increased significantly during flower bud differentiation and decreased dramatically after the completion of flower bud differentiation. In contrast, indoleacetic acid (IAA) showed the opposite effect, decreasing with increases in the flower bud differentiation rate but increasing significantly during the bolting process of Welsh onion (Su et al., 2007). It is reasonable to assume that IAA inhibits flower bud differentiation but improves plant bolting. Similarly, zeatin riboside (ZR) also showed an enhancing effect on plant bolting (Song et al., 2007). Guo (1996) reported that cytokinin (CTK) was a bulbing initiator but had no visible effect on bulb enlargement, while IAA and ethylene improved bulb formation. However, few studies have investigated the role of abscisic acid (ABA) on garlic bolting or bulbing.

Jasmonic acid (JA) and related compounds are widely distributed among higher plants (Meyer et al., 1984) and play important roles in the regulation of plant development (Koda, 1992). It was found that jasmonates were potent inducers of vegetative storage protein gene expression (Staswick, 1990) and proteinase inhibitors of defense proteins (Farmer and Ryan, 1990, 1992). It is generally believed that the bulbing process is regulated by the balance between the ‘bulbing hormones’ and GA (Koda, 1997). Ravnikar et al. (1993) reported that JA significantly enhanced the shoot and bulb development *in vitro* in concentrations from 1-10 µM and suggested that JA might play an important role in the formation of storage organs in plants, such as garlic bulbs. Nojiri et al. (1992) reported that by considering that bulbing was involved in the disruption of microtubules, jasmonic acid (JA) and methyl jasmonate (MeJA) were candidate bulbing hormones because of their microtubule-disrupting activities and wide distribution in higher plants. However, Li et al. (2000) reported that salicylic acid (SA) played an important role in garlic bulb formation and that MeJA likely enhanced the endogenous SA content of garlic plant, thus improving bulbing.

In this study, the effects of temperature and photoperiod on garlic growth, bolting and bulbing of three local cultivars were investigated in two consecutive years. Changes in the phytohormone and MeJA levels during this process were analyzed to identify the temperature and photoperiod requirements for garlic growth and development.

## RESULTS

### Main effect of cultivar/sown date, temperature and photoperiod treatments on garlic plant growth and development

The main effect of each factor was analyzed (Table 1-1, Table 1-2). All of the studied factors, including cultivar (C, 2012), temperature (T), photoperiod (L) and sown date (D, 2013), had a highly significant effect on plant standing height, BI, bolting rate, growth period and bulb weight in both years of the experiments (2012 and 2013). The higher test temperature (25°C) or longer photoperiod (14 h) presented a significant enhancing effect on the BI, bolting rate, maturity and bulb weight of garlic, while a lower temperature (15°C) resulted in the highest plant among the treatments (Table 1-1, Table 1-2). Cv. G107 had the highest BI, bolting rate and shortest growth period, while cv. G064 produced the highest plant standing height and bulb weight when sampled in 2012 (Table 1-1). For the sown dates, D_0820_ had a significant effect on all of the studied indicators, followed by D_0920_ (Table 1-2).

**Table 1-1.**
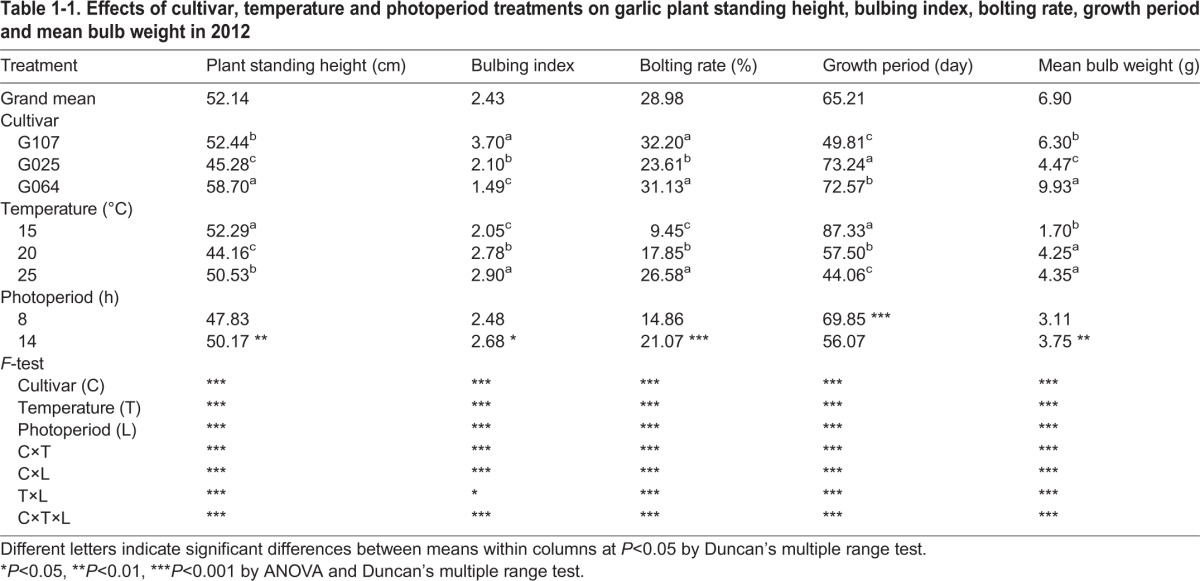
**Effects of cultivar, temperature and photoperiod treatments on garlic plant standing height, bulbing index, bolting rate, growth period and mean bulb weight in 2012**

**Table 1-2.**
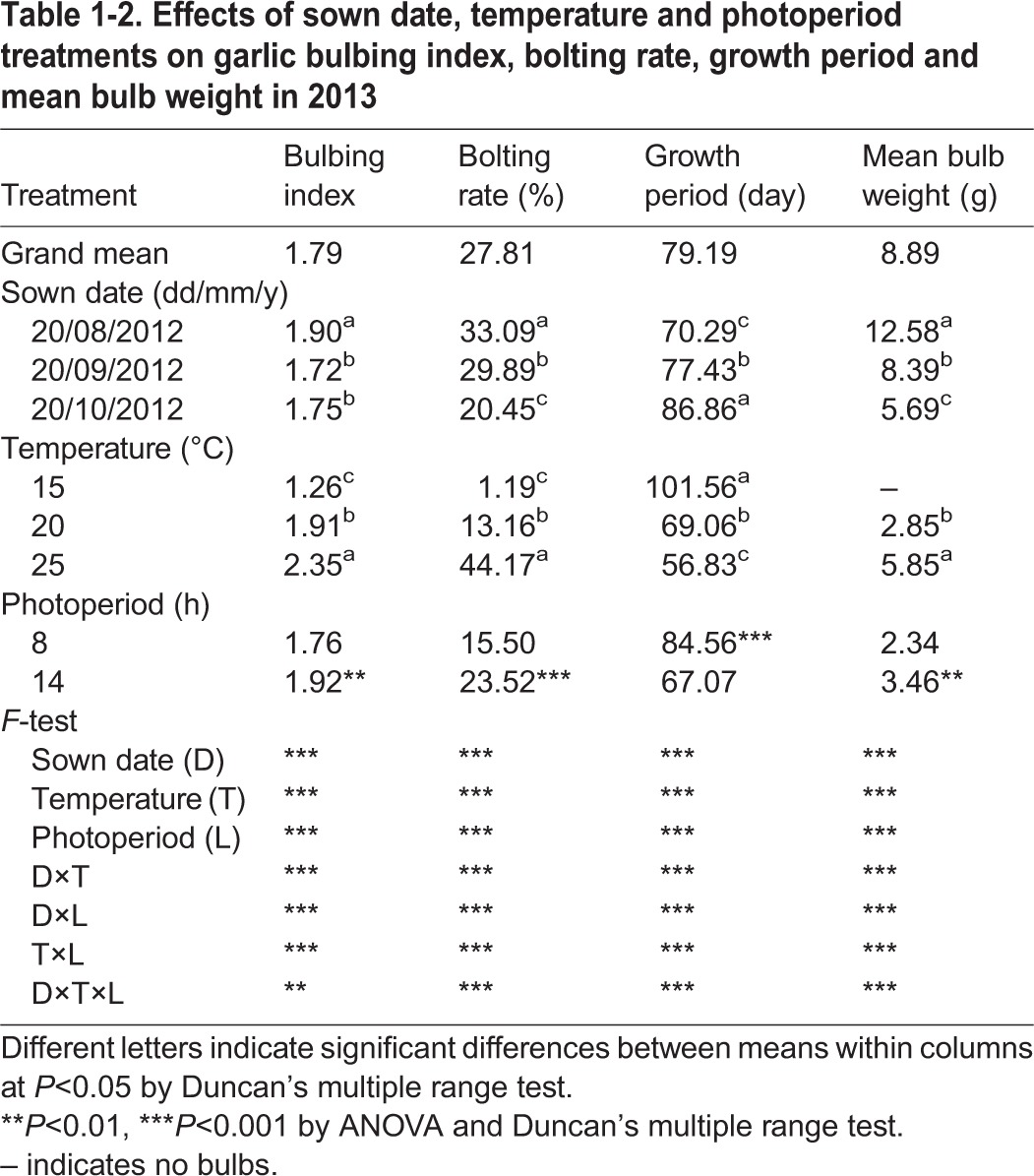
**Effects of sown date, temperature and photoperiod treatments on garlic bulbing index, bolting rate, growth period and mean bulb weight in 2013**

However, compared with plants grown in the field (T_ck_L_ck_), the bolting rate and bulb weight of all of the treated plants were significantly lower (Table S1-1, Table S1-2). It was obvious that the control plants had a much greater advantage in garlic growth and development than those grown in the growth chambers.

### Interaction effects of cultivar, temperature and photoperiod treatments on garlic standing height

**Fig. 1.**
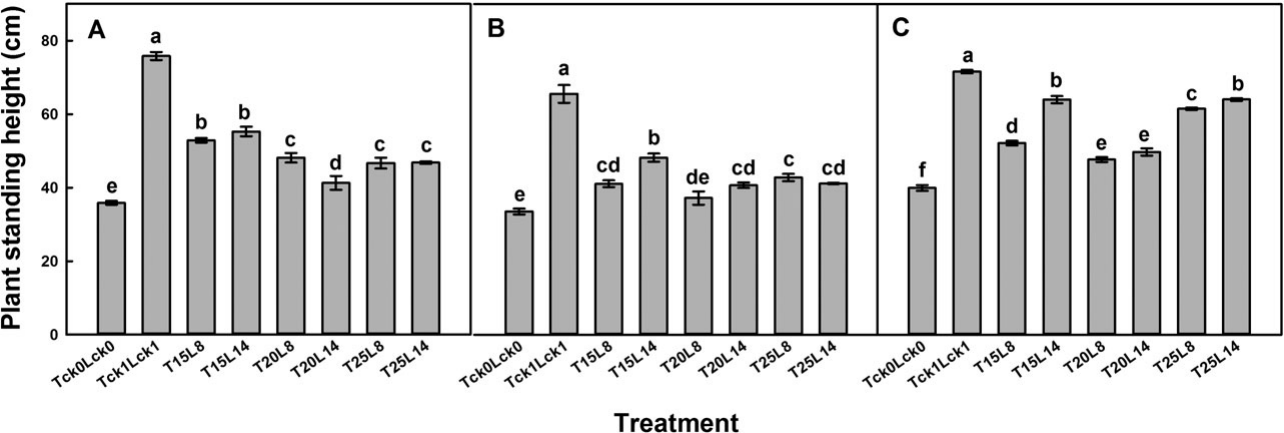
**Plant standing height of garlic.** Mean±s.e.m. heights of cvs G107 (A), G025 (B) and G064 (C) plants. Six plants of each block were taken with three replications. Different letters indicate significant differences at *P*<0.05 (ANOVA and Duncan's multiple range test), *n*=3.

The interaction effects of cultivar×temperature (C×T), cultivar×photoperiod (C×L) temperature×photoperiod (T×L) and C×T×L significantly influenced the plant standing height in 2012 (Table 1-1). It was shown in Fig. 1 that the growth of garlic plants differed significantly in response to different treatments when sampled 20 days after treatment in 2012 (Fig. 1). Although the plant standing height varied among cultivars, the same overall pattern was observed in which a longer photoperiod or lower temperature significantly promoted the standing height of the garlic plant among the treatments. The garlic plant growth responded favorably to the T_15/10_ treatment, especially T_15/10_L_14_, with increases of 54%, 43% and 60% for cvs G107, G025 and G064, respectively, compared with T_ck0_L_ck0_ (Fig. 1A-C). The lowest increase was observed in T_20/15_ plants with only a 15%, 10% and 21% increment for cvs.G107, G025 and G064, respectively. Moreover, a strong effect on garlic plant growth was evident for the longer photoperiod treatment (L_14_). However, note that the standing height of all of the treated plants was significantly inhibited compared with plants grown in the field (T_ck1_L_ck1_).

No significant effect was observed on the garlic pseudostem diameter and leaf number, indicating that the lower temperature treatment only promoted the longitudinal growth of the garlic plants (data not shown).

### Interaction effects of cultivar/sown date, temperature and photoperiod treatments on bulbing index

**Fig. 2.**
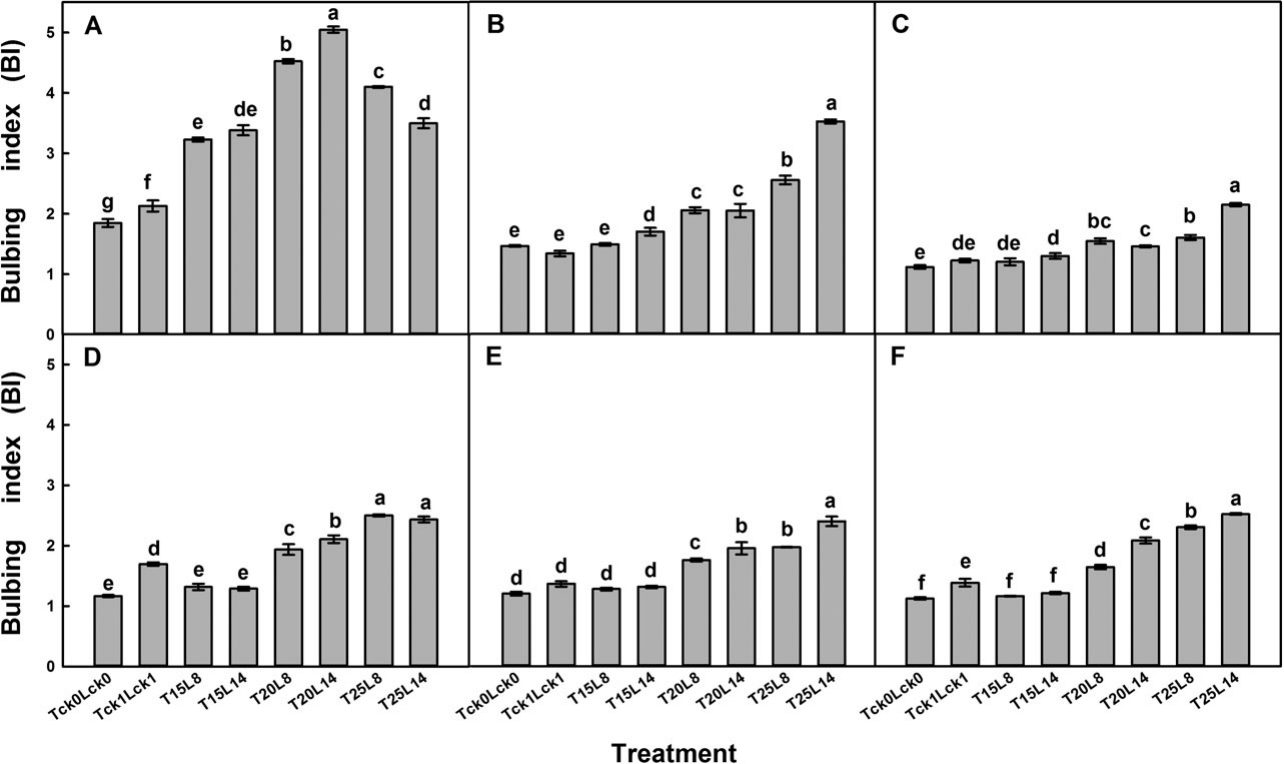
**Bulbing index of garlic plant.** (A) cv. G107, (B) cv. G025 and (C) cv. G064 in 2012. (D) sown on 20 August, (E) sown on 20 September and (F) sown on 20 October of cv. G064 in 2013. All data are presented as the mean±s.e.m. and six plants of each block were taken with three replications. Different letters indicate significant differences at *P*<0.05 (ANOVA and Duncan's multiple range test), *n*=3.

Bulbing was considered to start when BI=2. The interactions of C×T, C×L, sown date×temperature (D×T), sown date×photoperiod (D×L), T×L, C×T×L and D×T×L had a significant effect on BI in both years of the experiments (Table 1-1, Table 1-2). The results of the experiments from both years indicated that a higher temperature and longer photoperiod significantly enhanced the bulbing of garlic, whereas plants grown in the field (T_ck1_L_ck1_) had only a slight BI increase when sampled on 12 March 2012/2013 (Fig. 2). In 2012, after 20 days of treatment, bulbing began for cv. G107 in all of the treatments, and the highest BI was achieved under T_20/15_L_14_ (Fig. 2A). In contrast, the onset of bulb development only occurred in T_25/18_L_14_ for cv. G064 and T_20/15_/T_25/18_ for cv. G025; T_25/18_L_14_ had the highest BI for cvs.G025 and G064 (Fig. 2B,C).

The experiment in 2013 showed the same change trend in which the BI of all of the three sown date treatments responded favorably to the higher temperatures and longer photoperiod (T_25/18_>T_20/15_>T_15/10_; L_14_>L_8_; Fig. 2D-F). T_25/18_ L_14_ or D_0820_ produced the highest BI in cv. G064 plants. However, when treated under a lower temperature (T_15/10_), the BI of cv. G064 plants in both experimental years showed no significant increase compared with T_ck0_L_ck0_.

### Interaction effects of cultivar/sown date, temperature and photoperiod treatments on garlic bolting rate, growth period, rate of one-clove bulb and bulb characteristics

The interactions of C×T, C×L, D×T, D×L, T×L, C×T×L and D×T×L significantly affected the bolting rate, growth period and bulb weight of garlic in both years of the experiments (Table 1-1, Table 1-2).

Although the bolting rate of treated plants was far less than that grown in a field (T_ck_L_ck_; Table S1-1, Table S1-2), it was evident that the higher temperatures and longer photoperiod were required for the bolting of garlic (Table 2-1, Table 2-2). As shown in Table 2-1, the garlic bolting rate differed greatly among the cultivars. Cv. G107 bolted well even under the lower-temperature (T_15/10_) and shorter-photoperiod (L_8_) treatments, as evidenced by the bolting rate reaching 24.4%. In contrast, cvs G025 and G064 only slightly bolted under the T_15/10_ treatment; only cv. G064 bolted 9.3% under T_15/10_L_14_. As the temperature increased, the bolting rate of cv. G064 increased. T_25/18_L_14_ produced the highest bolting rate (51.1%) among all of the treatments. However, T_25/18_ was not suitable for the bolting of cv. G107 or cv. G025. T_20/15_L_14_ plants were recorded with the highest bolting rate for cvs G107 and G025 among all of the treatments, reaching 26.1% and 30.2%, respectively.

**Table 2-1.**
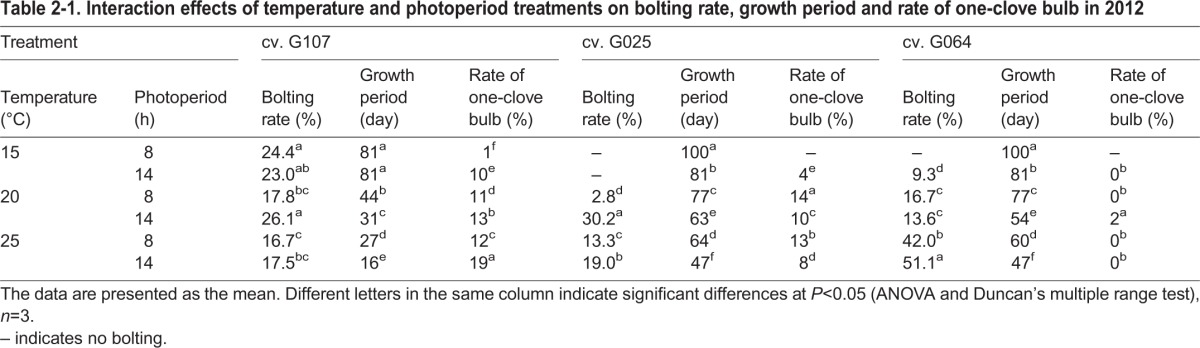
**Interaction effects of temperature and photoperiod treatments on bolting rate, growth period and rate of one-clove bulb in 2012**

**Table 2-2.**
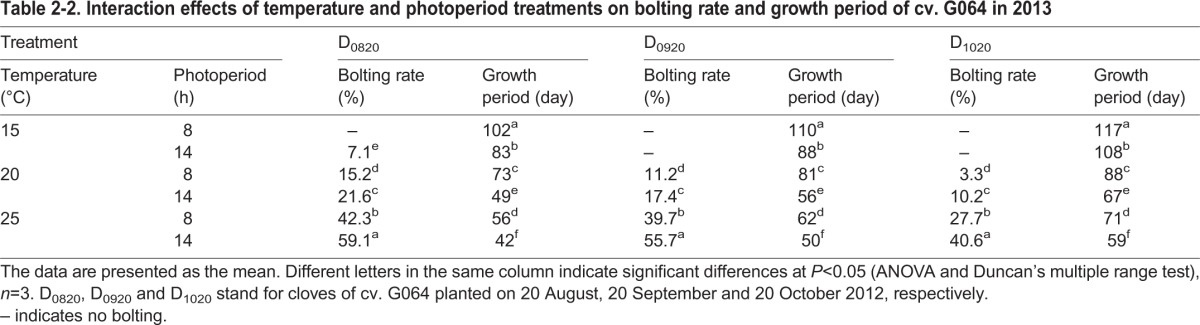
**Interaction effects of temperature and photoperiod treatments on bolting rate and growth period of cv. G064 in 2013**

A similar result of cv. G064 was obtained in the experiment in 2013 in which the bolting rate increased as the temperature and photoperiod increased in all of the three sown date treatments (Table 2-2). The highest bolting rate was recorded in T_25/18_L_14_ plants, reaching 59.1%, 55.7% and 40.6%, respectively, for the three sown date treatments, except for the control. When treated with 15°C, only D_0820_ had a lower bolting rate (7.1%).

Lower temperatures and shorter photoperiods resulted in a significant increase in the time to reach maturity for the garlic plants in all of the studied cultivars in both years (Table 2-1, Table 2-2). The overall growth period was longest under T_15/10_L_8_ in 2012 and 2013 and decreased for higher temperatures, longer photoperiods and older plant age; the growth period was shortest under T_25/18_L_14_ or D_0820_ (Table 2-1, Table 2-2; Table S1-1, Table S1-2). This finding suggested that higher temperatures and longer photoperiods enhanced the maturity of the garlic plant and shortened the growth period. One-clove bulbs were also harvested during the experiment. Cv. G107 produced the most one-clove bulbs, whereas cv. G064 barely produced any one-clove bulbs in both of the studied years.

The effect of temperature and photoperiod treatment on the bulb characteristics was cultivar-specific (Table 3-1), and plants grown in the field (T_ck_L_ck_) had the largest bulbs (Table S1-1, Table S1-2). Plants of cv. G107 produced bulbs regardless of temperature or photoperiod (Table 3-1). Only cv. G107 produced bulbs under T_15/10_L_8_, albeit very small ones. The bulbing responded favorably to treatments with higher temperatures and longer photoperiods. However, T_25/18_ suppressed the bulb size of cvs G107 and G025 compared with T_20/15_. Overall, except for the controls, T_20/15_L_14_ produced the largest bulbs for cvs G107 and G025 (Table 3-1), while T_25/18_L_14_ produced the best bulb characteristics for cv. G064.

**Table 3-1.**
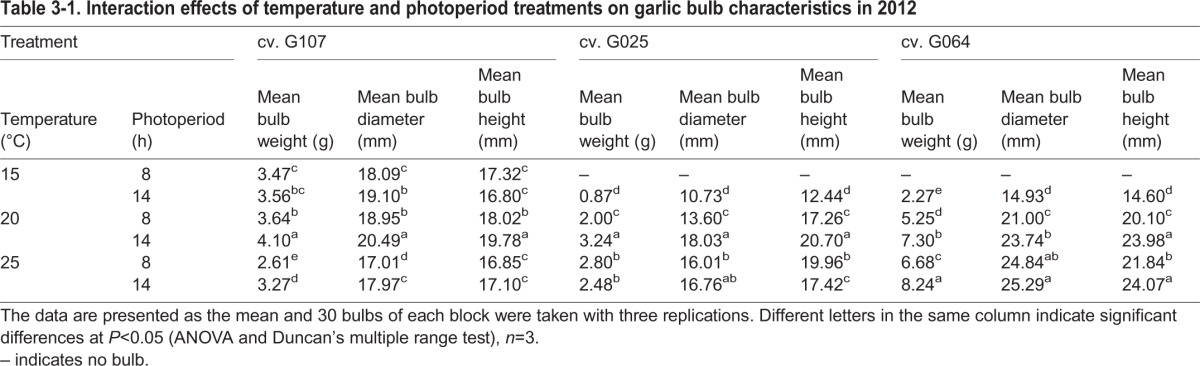
**Interaction effects of temperature and photoperiod treatments on garlic bulb characteristics in 2012**

In 2013, the bulb weight of cv. G064 increased as the temperature, photoperiod and plant age increased (Table 3-2). Plants of T_25/18_L_14_ were recorded as having the largest bulb, especially for D_0820_ plants, except for the controls. No bulbs were harvested for cv. G064 when treated with T_15/10_ in both 2012 and 2013.

**Table 3-2.**
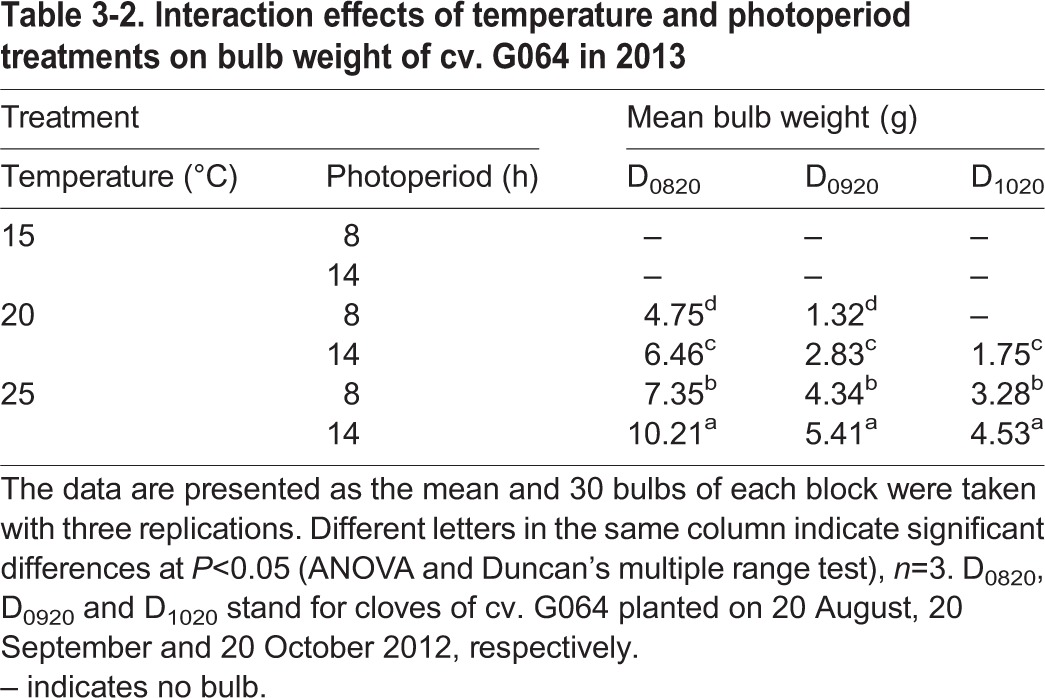
**Interaction effects of temperature and photoperiod treatments on bulb weight of cv. G064 in 2013**

### Main effect of cultivar, temperature and photoperiod treatments on endogenous phytohormone and MeJA level

The main effect of each factor on endogenous phytohormone and MeJA levels in garlic leaves was analyzed in 2012 (Table 4). A significant influence was recorded for all of the studied factors (C, T, and L). The responses were similar to BI and bolting rate.

**Table 4.**
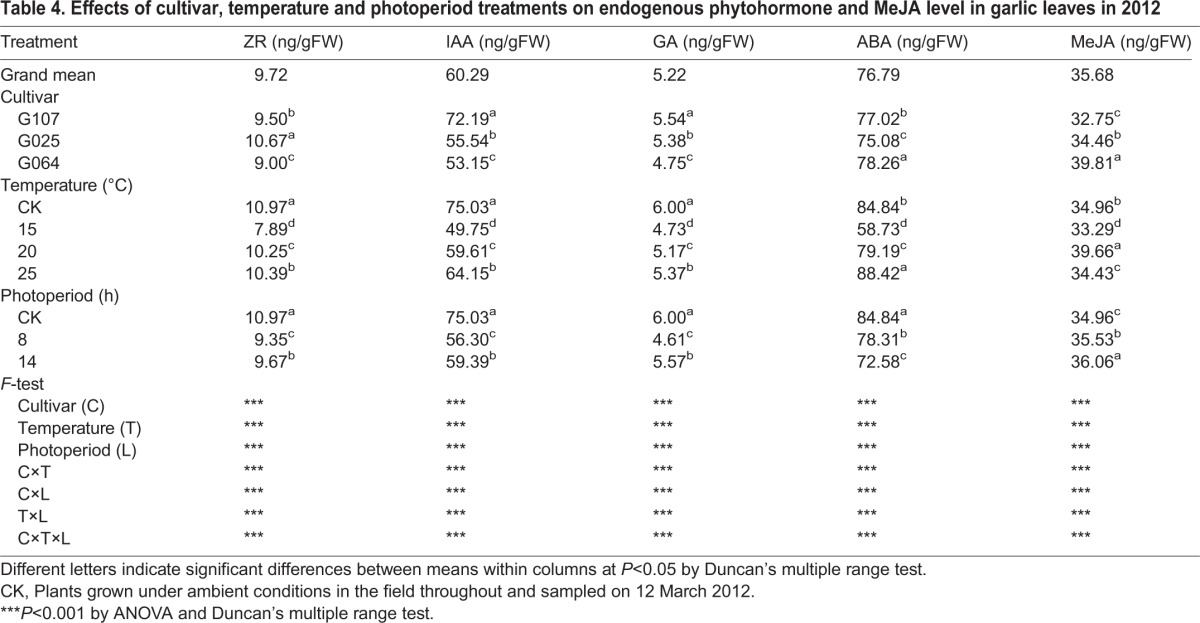
**Effects of cultivar, temperature and photoperiod treatments on endogenous phytohormone and MeJA level in garlic leaves in 2012**

Cv. G107, with the highest BI and bolting rate and earliest maturity, had the highest IAA and GA levels, while cv. G064, with the highest plant standing height and bulb weight and lowest BI, showed the highest ABA and MeJA levels and lowest ZR, IAA and GA levels. Cv. G025, with the lowest plant standing height, bolting rate, bulb weight and longest growth period, was recorded with the highest ZR level and lowest ABA levels.

Similar to the bolting rate and bulb weight, the temperature and photoperiod treatments significantly reduced the endogenous ZR, IAA, GA and ABA levels compared with the control (except for the ABA level in T_25/18_ plants; Table 4). Nevertheless, the higher temperature showed a significantly improving effect on the endogenous phytohormone (T_25/18_) and MeJA (T_20/15_) levels among the temperature treatments, which agreed with the change trend of the bolting rate and bulb weight; the lowest endogenous phytohormone and MeJA levels were achieved in T_15/10_ plants. In addition, T_25/18_, with the earliest maturity, had the highest ABA level. The photoperiod treatment showed a different influence in which the ZR, IAA, GA and MeJA levels were significantly higher in L_14_ plants than in L_8,_ while the ABA level was higher in L_8_ plants.

### Interaction effects of cultivar, temperature and photoperiod treatments on endogenous phytohormone and MeJA level

It is shown in Table 4 that the interactions of C×T, C×L, T×L and C×T×L significantly affected the endogenous phytohormone and MeJA levels in 2012.

**Fig. 3.**
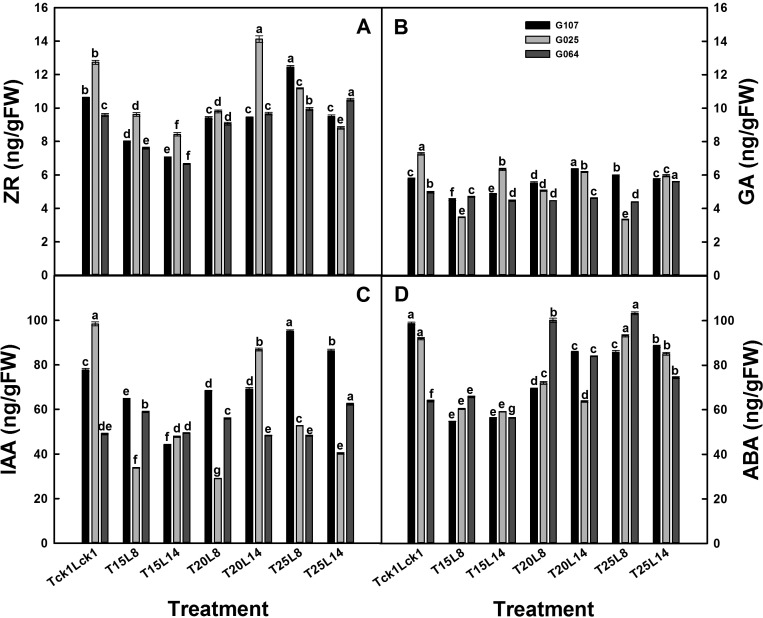
**Endogenous phytohormone level of garlic cvs G107, G025 and G064 in 2012.** (A) ZR, (B) GA, (C) IAA and (D) ABA. All data are presented as the mean±s.e.m. and six plants of each block were taken with three replications. Different letters indicate significant differences at *P*<0.05 (ANOVA and Duncan's multiple range test), *n*=3.

**Fig. 4.**
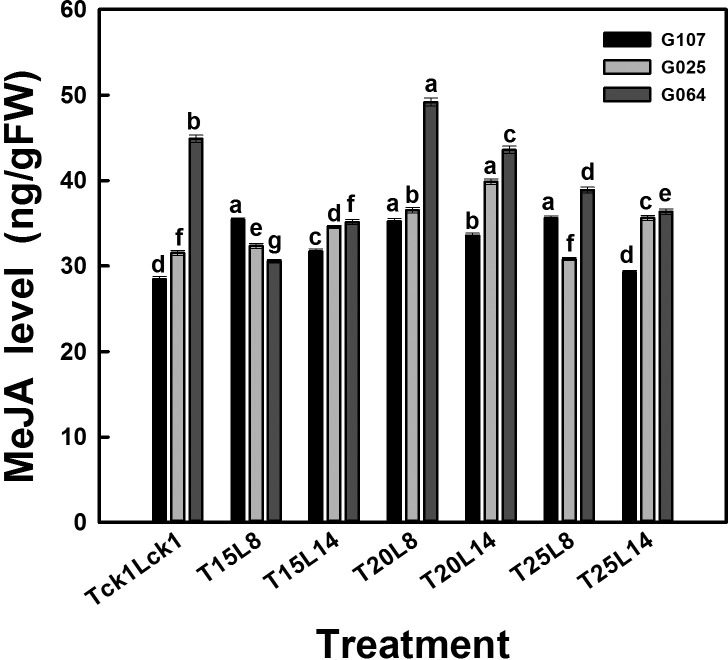
**MeJA level of garlic cvs G107, G025 and G064 in 2012.** All data are presented as the mean±s.e.m. and six plants of each block were taken with three replications. Different letters indicate significant differences at *P*<0.05 (ANOVA and Duncan's multiple range test), *n*=3.

The pattern for phytohormone and MeJA levels varied according to the cultivars (Figs 3, 4). After 20 days of treatment, the endogenous phytohormone levels were partially decreased, and the MeJA level was increased, except for cv. G064, compared with plants grown in the field.

For cv. G107, a significant reduction in the studied phytohormone was observed, especially when treated with T_15/10_. ZR and IAA behaved similarly: they decreased with the longer photoperiod and increased with the higher temperatures. Plants treated with T_15/10_L_14_ and T_25/18_L_8_ produced the lowest and highest levels, respectively. Similarly, GA and ABA responded synchronously in the leaves of cv. G107. The endogenous levels of GA and ABA increased with the higher test temperatures and longer photoperiod; the lowest levels were achieved in T_15/10_L_8_ plants. Moreover, the MeJA level in cv. G107 increased significantly after the 20-day treatment compared with plants grown in the field (T_ck1_L_ck1_); the shorter photoperiod had a significantly stronger enhancing effect as shown by L_8_ and T_20/15_ having the highest MeJA level in cv. G107.

Cv. G025 plants treated with T_20/15_L_14_ produced the highest phytohormone and MeJA levels among all of the treatments (except for ABA). This variation was in line with bolting rate and bulb weight. ZR and IAA levels were higher when treated with longer photoperiod (L_14_) or higher temperature (T_20/15_), which was different from cv. G107. GA responded similarly to that in cv. G107 in which it was significantly improved by a longer photoperiod (L_14_) or higher temperature (T_20/15_) treatment. However, ABA responded differently in cv. G025 from GA; the ABA level was significantly reduced by a longer photoperiod and was promoted by the highest test temperature (T_25/18_) treatment. After a 20-day treatment, the MeJA level in cv. G025 plants was significantly increased, especially when treated with a longer photoperiod (L_14_) or a higher temperature (T_20/15_) compared with the control (T_ck1_L_ck1_).

Plants of cv. G064 showed the same change trend as cv. G025 regarding the GA and ABA levels; the GA level increased with a longer photoperiod, while the ABA level decreased with a longer photoperiod. The highest phytohormone level was achieved in T_25/18_L_14_ plants (except for ABA). This variation was similar to that for the bolting rate. When treated at lower temperatures (T_15/10_ or T_20/15_), the ZR and IAA levels were significantly decreased with a longer photoperiod. The highest test temperature (T_25/18_) along with a longer photoperiod (L_14_) presented a significant enhancing effect. After 20 days of treatment, only cv. G064 plants treated with T_20/15_L_8_ produced an increased MeJA level; the MeJA level in cv. G064 plants of the remaining treatments was reduced compared with the control (T_ck1_L_ck1_). In contrast to the endogenous phytohormone in cv. G064, a higher test temperature (T_20/15_) along with a shorter photoperiod (L_8_) presented the strongest enhancing effect on the MeJA level.

Although the responses differed by cultivar, it was important to note that some of the phytohormone levels responded similarly in the different cultivars. IAA and ZR followed the same pattern in cv. G107 in which they were reduced with a longer photoperiod, while they increased with a higher temperature. A longer photoperiod significantly increased the GA levels in the three studied cultivars. ABA responded similarly in cvs G025 and G064 in which it decreased with a longer photoperiod. The MeJA levels in cvs G025 and G107 were significantly increased after 20 days of treatment compared with the control.

## DISCUSSION

The results presented in this study showed that garlic bolting and bulbing responded favorably to higher temperature and longer photoperiod treatments.

A variation among garlic cultivars in bolting, bulbing and responses to environmental signals is expected and is most likely similar to what common occurs in other *Allium* plants (Rabinowitch, 1985, 1990; Kamenetsky and Rabinowitch, 2002; Khokhar et al., 2007; Brewster, 2008; Dong et al., 2013). In alliaceous crops, bolting depends on environmental signals, i.e. high (tulip, narcissus) or low (onion) temperatures or long photoperiods (lily) (Kamenetsky and Rabinowitch, 2002; Khokhar et al., 2007; Brewster, 2008; Benschop et al., 2010; Dong et al., 2013). Takagi (1990) reported that low temperatures promote the floral development of garlic and that long photoperiods are essential for floral scape elongation. Normally, bolting-type garlic plants require 30-40 days under 0-4°C or 50-60 days under 10°C at the four-leaf age for vernalization (Song et al., 2010). After that period, a long photoperiod (≥13 h) and higher temperature (20°C) are required for the bolting and bulbing of garlic (Wu et al., 2015, 2016). The cultivars used in this research belong to the bolting type and are widely cultivated in Shaanxi Province, China, especially for cv. G064 due to its high yield of bulbs and scapes. In our last paper, the effect of the clove chilling treatment on the growth and development of cv. G064 was studied (Wu et al., 2015). In this study, we wanted to further demonstrate the temperature and photoperiod requirement of this cultivar. Therefore, in the second year of this study (2013), cv. G064 was chosen for studies on the sown date effect. In the present research, after the saturation of vernalization, garlic plants were subjected to various combinations of temperature and photoperiod treatments for the following bolting and bulbing. The results showed that three studied cultivars had a higher bolting rate under higher temperatures (20°C or 25°C) and with a 14-h photoperiod compared with all of the treatments in both experiments, which was in agreement with previous findings. However, the temperature requirement for bolting differed among cultivars: 20°C was critical for cvs G107 and G025, while cv. G064 bolted best at 25°C (Table 2-1). In 2013, three sown dates of cv. G064 were studied, and a similar result was obtained (Table 2-2). The results in 2013 provide further evidence for the improving effect of a higher temperature and longer photoperiod on garlic bolting. In addition, cv. G107 bolted even at 15°C/10°C, which was considered a low temperature in this study. It is reasonable to assume that longer photoperiods are indispensable for the bolting of garlic, whereas the temperature requirement varies by cultivar. According to the biological classification of garlic, cv. G107 belongs to the early maturing type, which bolts early, has earlier bulb formation and has an earlier harvest date compared with other cultivars; G025 is a middle maturing cultivar, while cv. G064 is one of the late maturing cultivars. The significantly different characteristics of the chosen cultivars might result in the different performances of bolting rate and bulb weight. As shown in Table 4, the endogenous phytohormone and MeJA levels were significantly different among the studied cultivars, which might explain the mechanism of the various bolting behaviors after temperature and photoperiod treatments (Figs 3, 4; Table 4).

It has long been accepted that day length and temperature play crucial roles in the formation and final size of the bulb. After 20 days of treatment, the bulbing process began for cv. G107 in all of the treatments. In contrast, the onset of bulb development only occurred at a higher temperature for cv. G025 and cv. G064. The results in 2013 were similar in which a higher temperature combined with a longer photoperiod significantly improved the bulb formation of garlic, especially for the older plants. The findings were in accordance with the previous study in which bulbing and bolting required higher temperatures and longer photoperiods (Brewster, 1977).

In this study, the three chosen cultivars were planted in the field simultaneously on 21 September 2011. As the garlic grew, the early maturing garlic (cv. G107) had an early onset of bolts and bulbs and completed the entire life circle early, thus explaining why when we transplanted the test plants into the growth chamber and then sampled them on 12 March, cv. G107 produced the largest BI, whereas cv. G064 had the lowest value (Table 1-1). Late maturing cv. G064 bolted late and had a late onset of bulbs. It is known that the late maturing cultivars have an advantage in yield and fruit quality partially due to the longer growth period. Furthermore, the biological characteristics of cvs G107 and G064 are different; the bulb weight of cvs G107 and G064 is 13-16 g and 55 g, respectively. Consequently, when harvested, cv. G064 had the largest bulb and highest yield.

After 20 days of growth, the control plants grown under ambient conditions showed only a slight bulbing index increment, which was identical to the plants treated under T_15/10_ (Fig. 2). However, after harvesting, the bulb weight and bolting rate of the control plants were significantly higher than all of the test plants (see Tables S1-1 and S1-2). The reason for this result might be that the mean temperature in March in the field was only 8°C, and the effective accumulated temperature (≥18°C) for bulbing was not sufficient when sampled on 12 March. Thus, the bulb enlargement process of the control plants was not activated. As the temperature increased and the photoperiod lengthened in the field, the superiority of the combined ambient conditions (temperature, photoperiod, sunlight, humidity, and atmosphere) became evident, leading to the highest bolting rate and largest bulb compared with the test plants. The results further revealed that the chosen cultivars have a good performance in bolting and bulbing (without any developmental disorders) during a years cultivation; the diverse behaviors of the test plants are due to the different treatment settings. After centuries of natural selection, garlic will grow better under natural temperature and photoperiod conditions in suitable zones. Thus, the growth data (bolting rate and bulb weight) of test plants grown in growth chambers are markedly lower than those grown in the field. Although we attempted to optimize the experimental design, the deficiency of the artificial environment is inevitable. The artificial light cannot compete with natural sunlight. Therefore, it is inappropriate to compare the growth data of plants grown in the growth chambers with those in the field. It is better to compare plant growth among plants grown in the growth chamber because they have the same growth conditions except for the studied factors (temperature and photoperiod). Moreover, based on the growth behaviors of garlic, the temperature and photoperiod range in this experiment is wide for the research of garlic bolting and bulbing. Thus, the paired comparisons between treatments are sufficient. We also discussed the growth of plants in the field to better elucidate the effect of our experiment. Our findings further demonstrate that only under controlled conditions can the critical condition or limiting factor for the development of different garlic cultivars be researched. Although the artificial environment is not sufficient for garlic development compared with the natural environment, the aim of this research is achieved; the higher temperature (25°C) and longer photoperiod (14 h) are significantly effective for garlic bolting and bulbing among all of the treatments, and the critical temperature and photoperiod conditions are cultivar specific.

It has been reported that garlic produces poor bulbs in warm, short-day lowland tropical regions, whereas in temperate zones, in which days are long and winters are cold, flower induction and differentiation occur and are often followed by scape elongation (Brewster, 2008). The findings of Mathew et al. (2010) showed that bolting in garlic had no adverse effect on the bulb weight. This study coincided with Mathew's findings in that the higher bolting rates and bulb yields occurred under the same treatment (T_20/15_L_14_ for cvs G107 and G025, T_25/18_L_14_ for cv. G064 in 2012 and 2013). It was assumed that under optimal growth conditions (especially suitable temperature and photoperiod conditions), bolting in garlic has no significantly repressive effect on bulbing and yield but improves the bulb formation of garlic (Mathew et al., 2010). It appears that competition for resources is a very important factor but only as a second or third step in a number of events (Mathew et al., 2010).

Burba and Riera (1997) observed a generally positive correlation between maturity and bulb yield in garlic. Panthee et al. (2006) also found that early maturity and high yields of garlic were related, motivating the breeding of a short-duration and high-yielding cultivar. The findings in this research are in accordance with these previous studies. Under higher temperature and long photoperiod conditions, the garlic plant matures earlier with a higher yield, which is of great benefit for the production of fresh garlic scape and bulb.

Bolting and bulbing are regulated by internal signals, which can be stimulated or inhibited by the environmental conditions. It had been widely reported that phytohormone regulated the plant growth, and JA was considered to play an important role in the formation of garlic bulb (Koda, 1997; Ravnikar et al., 1993). Our results showed that the control plants grew under field conditions throughout the years 2012 and 2013, with the highest bolting rate and bulb weight, produced the highest endogenous ZR, IAA and GA levels and the second highest endogenous ABA and MeJA levels compared with the test plants (Table 4). Furthermore, the endogenous phytohormone and MeJA levels in the test plants were significantly increased by the highest studied temperature (25°C, except for MeJA, which had highest level under 20°C) and longer photoperiod (14 h, except for ABA, which had highest level under 8 h) and was reduced by the lowest test temperature (15°C) and shorter photoperiod (8 h, except for ABA). This response coincided with that of the BI, bolting rate, growth period and bulb weight. Plants treated with T_20/15_L_14_ and T_25/18_L_14_ had the highest phytohormone levels (except for ABA) for cv. G025 and cv. G064, respectively, along with the best bolting and bulbing behavior and shorter growth period. The results further demonstrated that the higher bolting or bulbing ability and shorter growth period might be due to the higher endogenous phytohormone of plants. Nevertheless, plants with the lowest phytohormone and MeJA levels (treated at 15°C) presented the tallest standing height, except for the control. It seems that the higher endogenous phytohormone and MeJA levels significantly reduced the plant standing height of the three studied cultivars. The results partially illustrated that plant growth and development are complicated processes regulated by an intricate network of signaling pathways.

It was shown that the GA level was significantly increased by higher temperature and longer photoperiod in the three studied cultivars, which was in agreement with previous studies (King et al., 2006). The higher GA level might contribute to the higher bolting or bulbing ability under a longer photoperiod treatment. However, the previous research showed that GA inhibited the bulb formation (Koda and Okazawa, 1983; Xu et al., 1998). This may due to the different experimental conditions (temperature, photoperiod or cultivar used), which indicates that the endogenous GA level is regulated by the interactions between the environmental conditions and the cultivar.

Furthermore, the results showed that ABA and GA responded similarly to various treatments for cv. G017 in which they increased under the longer photoperiod or higher temperature treatment. The results were in agreement with the previous findings that ABA had a similar behavior to GA in the early stage of plant bolting (Song et al., 2007). The increased endogenous ABA and GA levels in cv. G107 plants might accelerate the maturing process of garlic plant, which leads to the shorter growth period under longer photoperiod and higher temperature conditions. However, when analyzing the effect of each factor and their interactions (Table 4), ABA responded differently from GA to the photoperiod length; the ABA level was increased by a shorter photoperiod (Table 4). In addition, as mentioned above, plants grown in the field had the highest endogenous phytohormone levels, except for ABA; T_25/18_ plants had an even higher ABA level than the controls along with the shortest growth period. It is assumed that higher ZR, IAA and GA levels significantly promote the garlic bolting and bulbing, whereas ABA acts more on the maturity of plants and is more sensitive to temperatures than photoperiods. The results illustrate that the growth of garlic plants is regulated by the interaction of endogenous phytohormones rather than the plant's hormonal action, and the significantly different phytohormone levels among cultivars might lead to the various responses to temperature and photoperiod treatments.

IAA and ZR were reported to have an enhancing effect on plant bolting (Song et al., 2007; Su et al., 2007). In our study, the IAA and ZR levels were increased as the test temperatures and photoperiods increased (Table 4). However, regarding the cultivar effect, this enhancing effect was only confirmed for cvs G025 and G064 (Fig. 3A,B). Cv. G107 responded adversely to the photoperiod treatment in which a longer photoperiod with a higher bolting rate produced lower IAA and ZR levels. The reason might due to the sampling dates. It is preferable to measure late in the period of flower bud differentiation in late-maturing cultivars and cultivars with low bolting or earlier in early-maturing cultivars and cultivars with high bolting; then, we might obtain the expected results.

MeJA was considered the ‘bulbing substance’. As shown in Table 4, the MeJA level was significantly increased with a higher temperature (20°C had the highest MeJA level) and longer photoperiod treatment along with the BI and bulb weight. Although a significant increase was observed for cv. G107 and cv. G025 compared with the control, only the MeJA level in cv. G025 plants responded in accordance with the bulbing behavior in that it was improved with a longer photoperiod. The MeJA level in cvs G107 and G064 plants had the opposite response to the bulbing behavior in that it was inhibited by a longer photoperiod. However, cv. G064 was recorded with the highest MeJA level and the largest bulb. The various responses in MeJA levels might explain the different BI between the controls and test plants on 12 March (Fig. 4). As shown in Fig. 2, on 12 March, the control plants had the lowest BI compared with the test plants of cvs G107 and G025, which might be due to the much higher MeJA levels in the treated plants of cvs G107 and G025 than the controls (Fig. 4). Moreover, the BI of the control plants was higher than that in the T_15/10_ plants of cv. G064, which might explain the higher MeJA level in the controls of cv. G064 (Fig. 4, T_ck1_L_ck1_). It seems that bulbing of garlic is complicated due to the numbers of signals participating in the process from triggering to onset to formation. MeJA might be the ‘bulbing substance’, which changes in line with the BI and bulb weight for certain cultivars, but for others, the improving effect might act only at the final stage of garlic plant growth. However, the stage on which MeJA functions or whether it works in combination with other hormones remains unknown. Further experiments with more test cultivars and detailed sampling dates are required to elucidate these answers.

Cultivars along with the environmental conditions (temperature and photoperiod) influence the endogenous phytohormone levels, which ultimately regulate garlic growth and development. It is reasonable to assume that endogenous phytohormone (especially gibberellic acid) and MeJA levels are highly related to the bolting and bulbing ability of garlic, and higher levels are beneficial for garlic bolting and bulbing.

### Conclusions

In conclusion, it was evident that a higher temperature and longer photoperiod were essential for garlic bolting and bulbing. The endogenous phytohormone and MeJA levels varied significantly after treatment, which might lead to the different responses of the three studied cultivars to combined temperature and photoperiod treatments. It is assumed that higher endogenous phytohormone (especially GA) and MeJA levels are beneficial for garlic bolting and bulbing, but these levels inhibited the garlic vegetative growth (reduced plant standing height). The critical condition is cultivar specific. Cvs G107 and G025 bolt well and have better bulb formation under the conditions of 20°C–14 h, while 25°C–14 h is critical for bolting and bulbing of cv. G064.

## MATERIALS AND METHODS

### Experimental site description

**Fig. 5.**
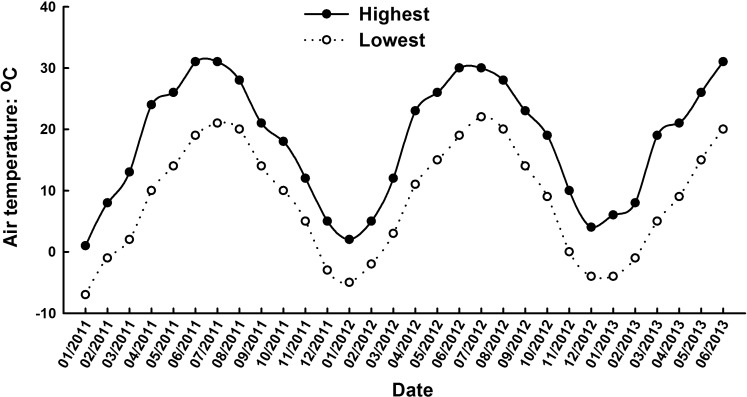
**Air temperature in the field in Yangling, Shaanxi Province, China from 2011-2013.** The data shows the mean of the highest or lowest temperature per day within a month.

**Fig. 6.**
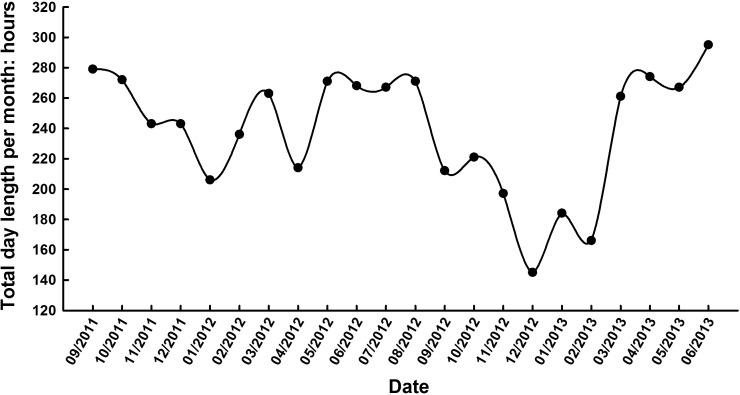
**Total day length per month in the field in Yangling, Shaanxi Province, China from 2011-2013.** The data shows the total hours of illumination intensity higher than 4 klux within a month.

The field experiments were carried out under a plastic tunnel at the Horticultural Experimental Station (34° 16′ N, 108° 4′ E) of Northwest A&F University, Yangling, Shaanxi Province, China in 2011-2012 and 2012-2013, respectively. The region has a subtropics monsoon climate, with hot summers and cool winters. The annual average temperature is 12.9°C, and the frost-free period is over 200 days. The air temperature and day length in the field from 2011-2013 are shown in Figs 5 and 6, respectively.

The chemical characteristics of the soil were as follows: pH (1:1 water) 7.83, electrical conductivity (1:5 soil/water) 239.1 µS cm^−1^, available nitrogen (N) 56.32 mg kg^−1^, available phosphorus (P) 52.57 mg kg^−1^ and available potassium (K) 224.90 mg kg^−1^.

Before clove planting, the plot was deeply ploughed and uniformly tilled. Next, 1.5 kg of ‘Pengdixin’ (organic matter≥30%, N+P+K≥4%, humic acid≥20%, organic sylvite≥5%; Zhengzhou, Henan Province, China), 0.25 kg of Stanley (21N-10P-11K; Yimeng, Shandong Province, China) and 0.4 kg of ammonium hydrogen carbonate (Hanzhong, Shaanxi Province, China) were broadcast and incorporated per plot as the base fertilizer prior to planting. Standard agricultural practices were applied throughout. The garlic plant morphological traits when transplanting are shown in Table 5-1.

**Table 5-1.**
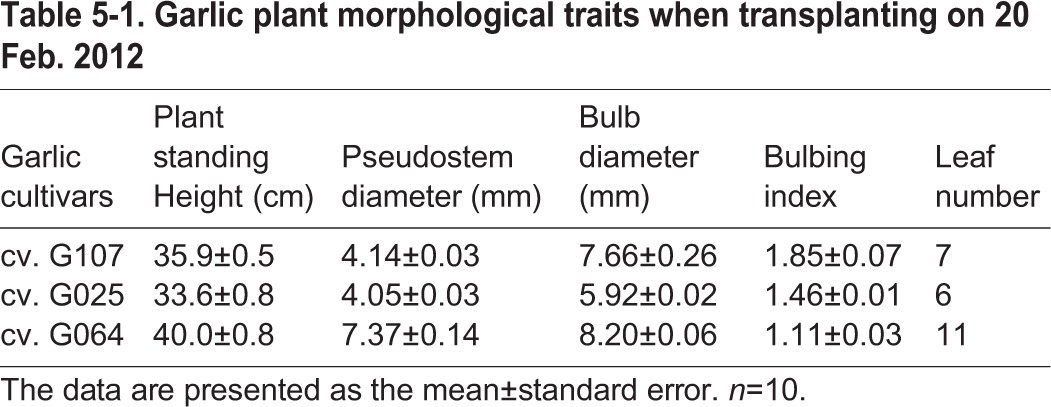
**Garlic plant morphological traits when transplanting on 20 Feb. 2012**

### Temperature and photoperiod treatments

The experiment in 2011-2012 was a three-factor (cultivar×temperature×photoperiod) randomized block experiment. Uniform and sound cloves of three local cultivated garlic cultivars (early mature cultivar: G107, middle mature cultivar: G025 and late mature cultivar: G064; all studied cultivars belong to the bolting types) were chosen as the material and were sterilized using 0.6% Dacotech (75% chlorothalonil, Syngenta, China). The phenotype of cv. G107 was as follows: plant standing height 74 cm, leaf number 12-13, bulb weight 13-16 g, bulb diameter 3-4 cm, 8-9 cloves arranged in two whorls (outer whorl 6 cloves, inner whorl 2-3 cloves), bolting rate 95%, growth period 210 days in overwinter planting and dormancy duration 35 days. The phenotype of cv. G025 was as follows: plant standing height 94 cm, leaf number 12-13, bulb weight 30 g, bulb diameter 4-5 cm, 10-11 cloves arranged in two whorls (no significant difference between the clove number of outer whorl and inner whorl), bolting rate 100%, growth period 250 days in overwinter planting and dormancy duration 40 days. The phenotype of cv. G064 was as follows: plant standing height 90 cm, leaf number 13-15, bulb weight 55 g, bulb diameter 5 cm, 12-13 cloves arranged in two whorls (outer whorl 6-7 cloves, inner whorl 5-7 cloves), bolting rate 80%, growth period 260 days in overwinter planting and dormancy duration 40 days.

Then, the cloves were planted in the field on 21 September 2011 at a depth of 5 cm so that the plant spacing was 5 cm and the row spacing was 20 cm under the plastic tunnel. The plot area was defined by a 1.5-m-wide and 3.5-m-long bed with six planting rows. Each cultivar was planted in a different plot.

Naturally vernalized plants of three garlic cultivars were transplanted into 15 cm×15 cm pots on 20 February 2012 and were cultured with organic substrate ‘Jiahui’ (organic matter 20%-25%, humic acid 8%-10%, pH 6.5-6.8; Liaocheng, Shandong Province, China). Each pot received four uniform garlic plants, and nine pots were used per treatment for each replication. Three replications were used for this experiment. Before treatment, the plants were conditioned for two days under 20°C/18°C (day/night) and 80% RH to minimize transplanting shock.

On 22 February 2012, the plants were subjected to various combinations of temperatures [T_15/10_, 15°C/10°C; T_20/15_, 20°C/15°C and T_25/18_, 25°C/18°C (day/night)] and photoperiods (L_8_, 8 h and L_14_, 14 h) in six separate growth chambers (Ningbo Jiangnan Instrument Factory, Zhejiang Province, China) with 105 μmol m^−2^ s^−1^ PAR (photosynthetic active radiation) and 70% RH (relative humidity) per replication. The control plants remained in the field throughout the experiment (T_ck_L_ck_).

Prior to (denoted as T_ck0_L_ck0_) and 20 days after the temperature and photoperiod treatment (12 March 2012), six plants from six pots from each treatment of each replication were randomly sampled (the control plants are denoted as T_ck1_L_ck1_). After recording the morphological traits, the third and fourth leaves (from top) of the samples were rinsed with distilled water and dried with absorbent paper. The homogeneously mixed samples were then immersed in liquid nitrogen for several seconds and stored at −80°C in a freezer until analysis of the endogenous phytohormone and MeJA levels. From April 15 to May 2012, the garlic bolting rate, garlic bulb weight and garlic bulb characteristics were recorded.

A similar experiment was carried out in the following autumn in 2012-2013 as a three-factor (sown date×temperature×photoperiod) randomized block experiment with three replications. Uniform cloves of cv. G064 were planted on 20 August, 20 September and 20 October 2012; these dates corresponded to the sown date treatments of D_0820_, D_0920_ and D_1020_, respectively. On 22 February 2013, the plants of the three sown date treatments were subjected to various combinations of temperature [T_15/10_, 15°C/10°C; T_20/15_, 20°C/15°C and T_25/18_, 25°C/18°C (day/night)] and photoperiod (L_8_, 8 h and L_14_, 14 h) in six separate growth chambers (Ningbo Jiangnan Instrument Factory, Zhejiang Province, China) with 105 μmol m^−2^ s^−1^ PAR and 70% RH per replication. The garlic plant morphological traits when transplanting are shown in Table 5-2. The rest settings and management were the same as that for the experiment in 2012. From April 15 to May 2013, the garlic bolting rate and garlic bulb weight were recorded.

**Table 5-2.**
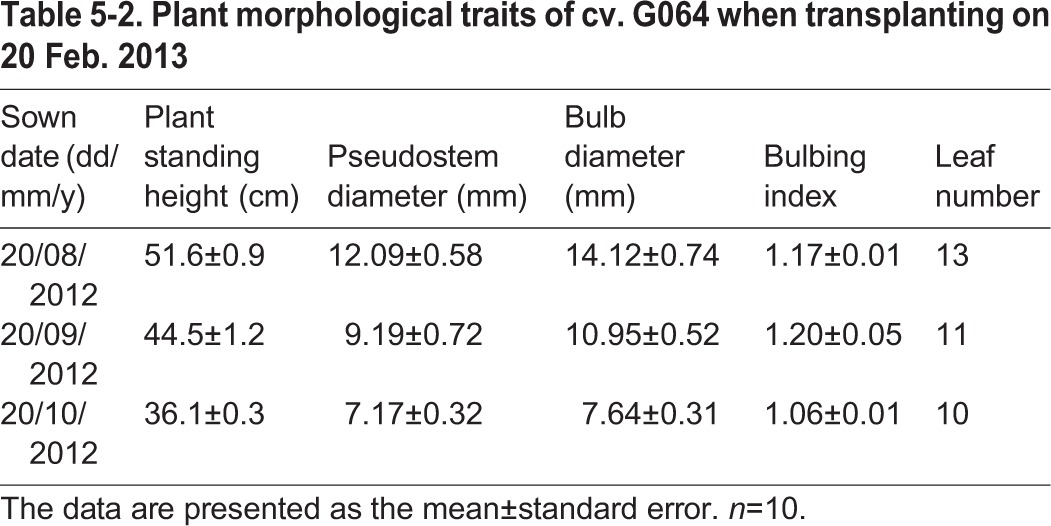
**Plant morphological traits of cv. G064 when transplanting on 20 Feb. 2013**

### Measurement of garlic plant morphologic traits and bulbing index

The garlic plant standing height, pseudostem diameter and bulb diameter were evaluated in the laboratory using a measuring tape (0.01 cm for plant standing height) and electronic vernier caliper (0.01 mm for pseudostem and bulb diameter). The number of plant leaves was counted by visual observation.

The bulbing index (BI) was expressed as the ratio of the bulb diameter to pseudostem diameter (Mann, 1952). Bulbing was considered to start when BI=2.

### Evaluation of garlic bolting rate, growth period, rate of one-clove bulb and bulb characteristics

The bolting rate was calculated as the ratio of scape number to the remaining number of garlic plants (30).

The harvest date was determined by the yellowing and partial drying of the canopy and/or leaf senescence. Then, the number of days of the growth period was calculated from the date of transplanting in growth chambers (22 February) to the harvest date.

After harvesting, the plants were subjected to curing at room temperature (22±2°C) for approximately 2 weeks, and then, the percentages of one-clove bulbs were recorded (Resende et al., 2011).

The bulb characteristics were recorded using the remaining bulbs (30).

### Evaluation of the endogenous phytohormone and MeJA content

The extraction, purification and determination of the endogenous IAA, ZR, ABA, GA and MeJA levels were performed using an indirect enzyme-linked immunosorbent assay (ELISA) technique, as described in previous publications (Yang et al., 2001a,b; Teng et al., 2006; Zhu et al., 2005; Zhao et al., 2006). The ELISA kit was provided by the College of Agriculture and Technology, China Agricultural University.

The samples (0.500 g) were homogenized in liquid nitrogen and ground in an ice-cooled mortar in 8 ml 80% (v/v) methanol extraction medium with butylated hydroxytoluene (1 mM) as an antioxidant. The extract was incubated at 4°C for 4 h and then centrifuged at 1150 ***g*** for 8 min at 4°C. The supernatants were passed through a C_18_ Sep-Pak cartridge (Waters, Milford, MA, USA). The efflux was collected and dried under N_2_. The residues were then dissolved in 2 ml 0.01 mM phosphate buffer saline (PBS) containing 0.1% (v/v) Tween 20 and 0.1% (w/v) gelatin (pH 7.5) to determine the levels of ZR, IAA, GAs, ABA and MeJA.

The mouse monoclonal antigens and antibodies against ZR, IAA, GAs, ABA and MeJA used in ELISA were produced at the Phytohormones Research Institute (China Agricultural University). ELISA was performed on a 96-well microtitration plate. Each well in the plate was coated with 50 µl coating buffer (1.5 g l^−1^ Na_2_CO_3_, 2.93 g l^−1^ NaHCO_3_, and 0.02 g l^−1^ NaN_3_, pH 9.6) containing 0.25 µg ml^−1^ antigens against the hormones. Ovalbumin solution (10 mg ml^−1^) was added to each well to block nonspecific binding. The coated plates were incubated for 30 min at 37°C. After washing four times with PBS containing 0.1% (v/v) Tween 20 buffer (pH 7.5), each well was filled with 100 µl of either garlic plant extracts or ZR, IAA, GAs, ABA and MeJA standards (0-100 ng ml^−1^ dilution range for ZR, IAA and ABA, 0-50 ng ml^−1^ for GA, 200 ng ml^−1^ for MeJA), and 50 µl of 20 µg ml^−1^ antibodies against ZR, IAA, GAs, ABA and MeJA. The antibodies against ZR, IAA, GAs, ABA and MeJA were obtained as described by Weiler et al. (1981).The plates were incubated for 30 min at 37°C, and were washed according to the above procedure. Then, 100 µl of a color-appearing solution containing 1.5 mg ml^−1^ 0-phenylenediamine and 0.04% (v/v) H_2_O_2_ was added to each well. The reaction progress was stopped by the addition of 50 µl 12 mM H_2_SO_4_ per well when the 100 ng ml^−1^ standard displayed a pale color and when the 0 ng ml^−1^ standard displayed a deep color in the wells. Color development in each well was detected using an ELISA Reader (model EL310, Bio-TEK, Winooski, VT) at optical density *A*490. ZR, IAA, GAs, ABA and MeJA contents were calculated following Weiler et al. (1981). The results are the means±s.e.m. of at least four replicates. In this study, the percentage recovery of each hormone was calculated by adding known amounts of standard hormone to a split extract. Percentage recoveries were above 90%, and all of the sample extract dilution curves paralleled the standard curves, indicating the absence of nonspecific inhibitors in the extracts.

### Statistical analysis

The data were subjected to analysis of variance (ANOVA) as a 3×3×2 (cultivar×temperature×photoperiod) factorial structure for the experiment in 2011-2012 and a 3×3×2 (sown date×temperature×photoperiod) factorial structure for the experiment in 2012-2013, using the SPSS 17.0 software package. Mean separations among treatments were performed using Duncan's multiple range tests at *P*<0.05.
